# Prognostic significance of HS2ST1 expression in patients with hepatocellular carcinoma

**DOI:** 10.1007/s13258-024-01556-0

**Published:** 2024-08-17

**Authors:** Ting Ting Chung, Sang Kyum Kim, Seung Jin Lee

**Affiliations:** College of Pharmacy, Chungnam National University, 99 Daehak-ro, Yuseong-gu, Daejeon, 34134 Republic of Korea

**Keywords:** Heparan sulfate sulfotransferase, Prognosis, Hepatocellular carcinoma, HS2ST1, TCGA, ICGC, GSEA

## Abstract

**Background:**

Heparan sulfate 2-*O*-sulfotransferase 1 (HS2ST1) catalyzes the sulfation of glucuronic acid residues in heparan sulfate proteoglycans, enabling these proteoglycans to interact with numerous ligands within tumor microenvironments. However, the prognostic role of HS2ST1 expression in cancer remains unclear.

**Objective:**

This investigated HS2ST1 expression levels and their prognostic significance in various cancer types, demonstrated the prognostic value of HS2ST1 expression in hepatocellular carcinoma (HCC) patients, and identified molecular signatures associated with *HS2ST1* expression.

**Methods:**

HS2ST1 expression and patient survival data from The Cancer Genome Atlas (TCGA) datasets were analyzed using the Gene Expression Profiling Interactive Analysis (GEPIA) portal. We obtained gene expression and clinicopathological information on HCC patients from the TCGA and the Japan and France International Cancer Genome Consortium (ICGC) databases and performed survival analyses. We also examined relevant protein networks, differentially expressed genes, gene set enrichments, and tumor immune microenvironment features associated with *HS2ST1* expression.

**Results:**

*HS2ST1* exhibited higher expression in eight tumor types compared with normal tissues and was associated with poor prognoses in five tumors, including HCC. HS2ST1 status correlated with poor prognosis in two ICGC HCC cohorts. Elevated *HS2ST1* expression in HCC tumors was associated with signaling pathways involved in cell cycle progression, protein secretion, and mTORC1 signaling. Moreover, *HS2ST1* expression levels were inversely correlated with immune cell infiltration in the tumor microenvironment.

**Conclusion:**

Our study elucidates the prognostic significance of HS2ST1 expression in HCC patients and provides insights into the potential roles of HS2ST1 in signaling pathways and the tumor microenvironment.

## Introduction

The tumor microenvironment comprises a heterogeneous population of proliferating tumor cells, infiltrating immune cells, and fibroblasts; extracellular matrix (ECM); a basement membrane; vasculature; and secreted factors (Sarrazin et al. 2011). Heparan sulfate proteoglycans (HSPGs) are essential for ECM assembly and signaling in such microenvironments. HSPGs are glycoproteins consisting of core proteins covalently attached to heparan sulfate chains, thus categorizing them as glycosaminoglycans. The 13 core HSPG proteins are distributed across various cellular compartments, including cell and basement membranes, the ECM, and secretory vesicles. HSPGs bind cytokines, chemokines, and growth factors; the bound materials serve as reservoirs from which regulatory factors can be released via selective degradation of the heparan sulfate chains. HSPGs also function as receptors for proteases and protease inhibitors. Membrane HSPGs, in combination with integrin and adhesion molecules, facilitate cell-ECM and cell–cell interactions; they serve both as co-receptors of various growth factor receptors and as endocytic receptors that clear bound ligands.

Most interactions between HSPGs and protein ligands occur through the heparan sulfate domains of HSPGs, although some ligands bind directly to the core proteins. The biosynthesis of heparan sulfate chains begins with posttranslational modifications in the Golgi apparatus upon delivery of the core proteins from the endoplasmic reticulum (De Pasquale and Pavone 2020). Tetrasaccharide linkers and the first *N*-acetyl-d-glucosamine residues are attached to the serine residues of core proteins. Subsequently, d-glucuronic acid and *N*-acetyl-d-glucosamine residues are alternately added to growing chains. Concurrently, each chain undergoes sequential modifications that include deacetylation, epimerization of d-glucuronic acid to l-iduronic acid residues, and sulfation. *N*-deacetylase/*N*-sulfotransferase 1–4 (NDST1-4) mediates the addition of sulfate to the free amino groups of *N*-acetyl-d-glucosamine residues from which acetyl groups have been removed. Heparan sulfate 2-*O*-sulfotransferase 1 (HS2ST1) catalyzes 2-*O*-sulfation at iduronic acid and, less frequently, glucuronic acid residues in heparan sulfate chain. HS6ST1-3 and HS3ST1-6 facilitate 6-*O* and 3-*O* sulfation at glucosamine residues, respectively. These modified domains appear in clusters of varying lengths, interspersed with unaltered segments; the domains create binding sites for protein ligands such as antithrombin, fibroblast growth factor (FGF), and the FGF receptor.

Uronyl 2-*O* sulfotransferase 1 (also known as HS2ST1) is the only enzyme that catalyzes the 2-*O* sulfation of iduronic acid residues in heparan sulfate chains. The perinatal lethality of *Hs2st1*-deficient mice is characterized by bilateral renal agenesis and defects of the eyes and skeleton (Kuehn et al. 2021). Liver-specific alterations in mouse *Hs2st1* levels affect lipoprotein clearance (Anower et al. 2019). However, the role of HS2ST1 in cancer remains poorly understood and appears to vary by cancer type. *HS2ST1* exhibited higher expression in osteosarcoma compared with normal tissue, but lower tumor expression levels correlated with poor prognosis in high-risk patients (Huang et al. 2022; Yang et al. 2022). HS2ST1 expression was associated with favorable prognoses in multiple myeloma patients (Bret et al. 2009). In contrast, breast cancer patients with high *HS2ST1* expression had a worse prognosis than those with lower *HS2ST1* expression (Kuehn et al. 2021; Teixeira et al. 2020; Kumar et al. 2020). Because the sulfation pattern is typically determined by the cell type in which heparan sulfate is expressed (Sarrazin et al. 2011), it is crucial to study the clinical significance of HS2ST1 expression in relation to the specific tumor of interest.

In this study, we investigated HS2ST1 expression and the prognostic value of *HS2ST1* expression across The Cancer Genome Atlas (TCGA) cohorts. We discovered that *HS2ST1* expression correlated with poor prognosis in the TCGA-Liver Hepatocellular Carcinoma (LIHC) cohort and was prognostically relevant in two independent International Cancer Genome Consortium (ICGC) cohorts of hepatocellular carcinoma (HCC) patients. Further analyses of the protein–protein networks and gene sets involved, as well as immune cell infiltration, provided insights into the biological processes affected by *HS2ST1* expression.

## Materials and methods

### Data collection and preprocessing

TCGA datasets from the Gene Expression Profiling Interactive Analysis (GEPIA) portal (http://gepia2.cancer-pku.cn/#index) were used to analyze differences in HS2ST1 expression levels between normal and tumor tissues, and patient prognoses across different cancers (Tang et al. 2019). Gene expression and clinical data from the TCGA-LIHC and ICGC databases of Japan and France (ICGC-LIRI-JP and ICGC-LICA-FR, respectively) were also collected and analyzed. The TCGA sample data were acquired using the UCSC Xena Browser (http://xena.ucsc.edu/) (Goldman et al. 2020). After removal of samples lacking survival data, information on 365 primary solid tumor samples was used in subsequent analyses. The ICGC cohort data were downloaded (https://dcc.icgc.org/) (Zhang et al. 2019) and curated using the same approach. Data from 231 Japanese and 98 French primary tumor samples were subjected to further analyses. RNA-seq data were preprocessed and normalized using DESeq2 software (Love et al. 2014).

### Survival analysis

Patients were stratified into *HS2ST1*-high- and -low-expression groups based on cutoffs determined using the maximally selected rank statistics of the Survminer package (version 0.4.9). This method yields an optimal cutoff point for evaluating overall survival. Kaplan–Meier (KM) analysis was performed and log-rank tests were conducted to evaluate the associations between different *HS2ST1* expression levels and overall survival in HCC patients.

### Protein network, differentially expressed gene (DEG), and gene set enrichment analyses

Protein–protein interaction analysis utilized the Search Tool for the Retrieval of Interacting Genes/Proteins (STRING) database (version 12) (Szklarczyk et al. 2023) to identify interactions between HS2ST1 and other proteins. This analysis provided insights into the roles and potential protein partners of HS2ST1 in molecular pathways, enhancing the overall understanding of HS2ST1 biological functions and their relevance to various cellular processes.

To identify HCC biological pathways involving HS2ST1, DEGs between the high- and low-*HS2ST1* expression groups of the TCGA cohort were identified using DESeq2 software (Love et al. 2014); the criteria were an adjusted P-value < 0.05 and an absolute log_2_-fold change of 1. Gene set enrichment analysis (GSEA) software version 4.3.2 was used to compare the high‐ and low‐HS2ST1-expression groups (Mootha et al. 2003; Subramanian et al. 2005). The hallmark gene set of the Molecular Signatures Database was utilized in the analysis (Liberzon et al. 2011, 2015; Subramanian et al. 2005).

### Links between tumor immunity and *HS2ST1* expression

The Estimation of STromal and Immune cells in MAlignant Tumor tissues using Expression data (ESTIMATE) algorithm (Yoshihara et al. 2013) was used to explore the relationship between *HS2ST1* expression levels, the tumor microenvironment, and the extent of tumor immune cell infiltration. Subsequently, the Cell-type Identification By Estimating Relative Subsets Of RNA Transcripts (CIBERSORT) algorithm (Newman et al. 2015) was applied to determine the relative scores of 22 different immune cells in the TCGA-LIHC cohort. Additionally, the TISIDB database (Ru et al. 2019), an integrated repository of tumor-immune system interactions, was utilized to investigate the relationship between immunomodulator levels and *HS2ST1* mRNA expression levels.

### Statistical analysis

All statistical analyses were conducted within R (version 4.4.0) and RStudio (version 2024.04.0) softwares. Survival analyses were performed using the R packages ‘Survival’ (version 3.5–8) and ‘Survminer’ (version 0.4.9). The significance threshold for all tests was set to P < 0.05.

## Results

### *HS2ST1* expression levels in various cancers

**Fig. 1:**
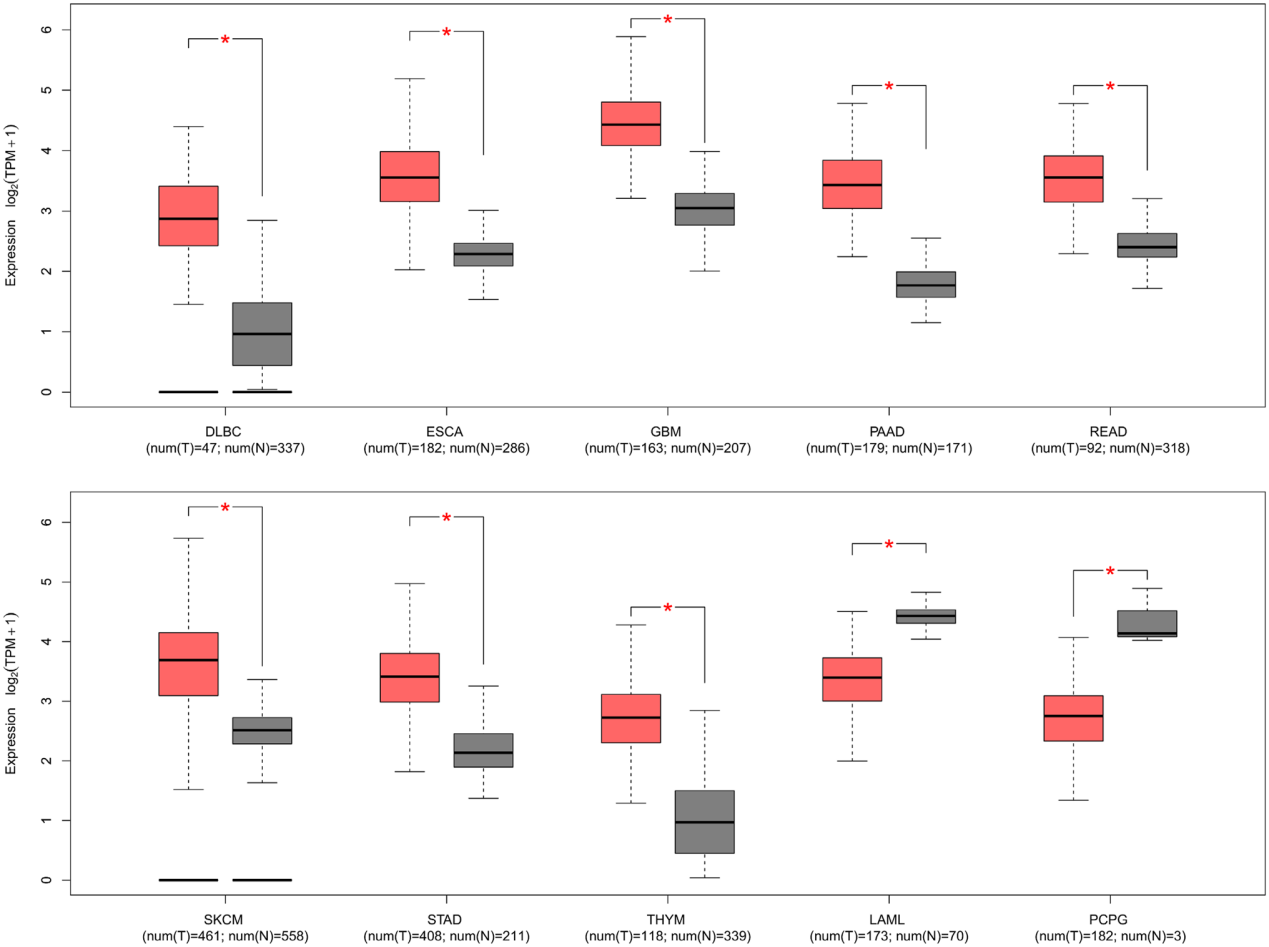
*HS2ST1* expression levels in tumor and normal tissues. *HS2ST1* expression levels [log_2_(TPM + 1) values] in tumor tissues and adjacent normal tissues of the TCGA datasets and normal tissues of the GTEx dataset (31 different tissue types) were analyzed using the GEPIA2 portal. Only cancers exhibiting significant differences (^*^: *P* < 0.05) in *HS2ST1* expression between tumor and normal tissues are shown. *DLBC* diffuse large B-cell lymphoma, *ESCA* esophageal carcinoma, *GBM* glioblastoma multiforme, *PAAD* pancreatic adenocarcinoma *READ* rectal adenocarcinoma, *SKCM* skin cutaneous melanoma, *STAD* stomach adenocarcinoma, *THYM* thymoma, *LAML* acute myeloid leukemia, *PCPG* pheochromocytoma and paraganglioma, *num(T)* number of TCGA tumor samples, *num(N)* number of normal TCGA and GTEx samples

*HS2ST1* expression levels in tumors were compared with those in adjacent normal tissues of the TCGA datasets and normal tissues of the GTEx dataset across various tissue types in the GEPIA2 portal. *HS2ST1* expression levels differed among cancer types (Fig. 1). Of the 31 cancers studied, *HS2ST1* expression levels in tumor tissues were higher (compared with normal tissue) in patients with diffuse large B-cell lymphoma (DLBC), esophageal carcinoma (ESCA), glioblastoma multiforme (GBM), pancreatic adenocarcinoma (PAAD), rectal adenocarcinoma (READ), skin cutaneous melanoma (SKCM), stomach adenocarcinoma (STAD), and thymoma (THYM). However, the reverse was observed in patients with acute myeloid leukemia (LAML) and pheochromocytoma and paraganglioma (PCPG).

### Prognostic value of *HS2ST1* expression in TCGA cohorts

**Fig. 2:**
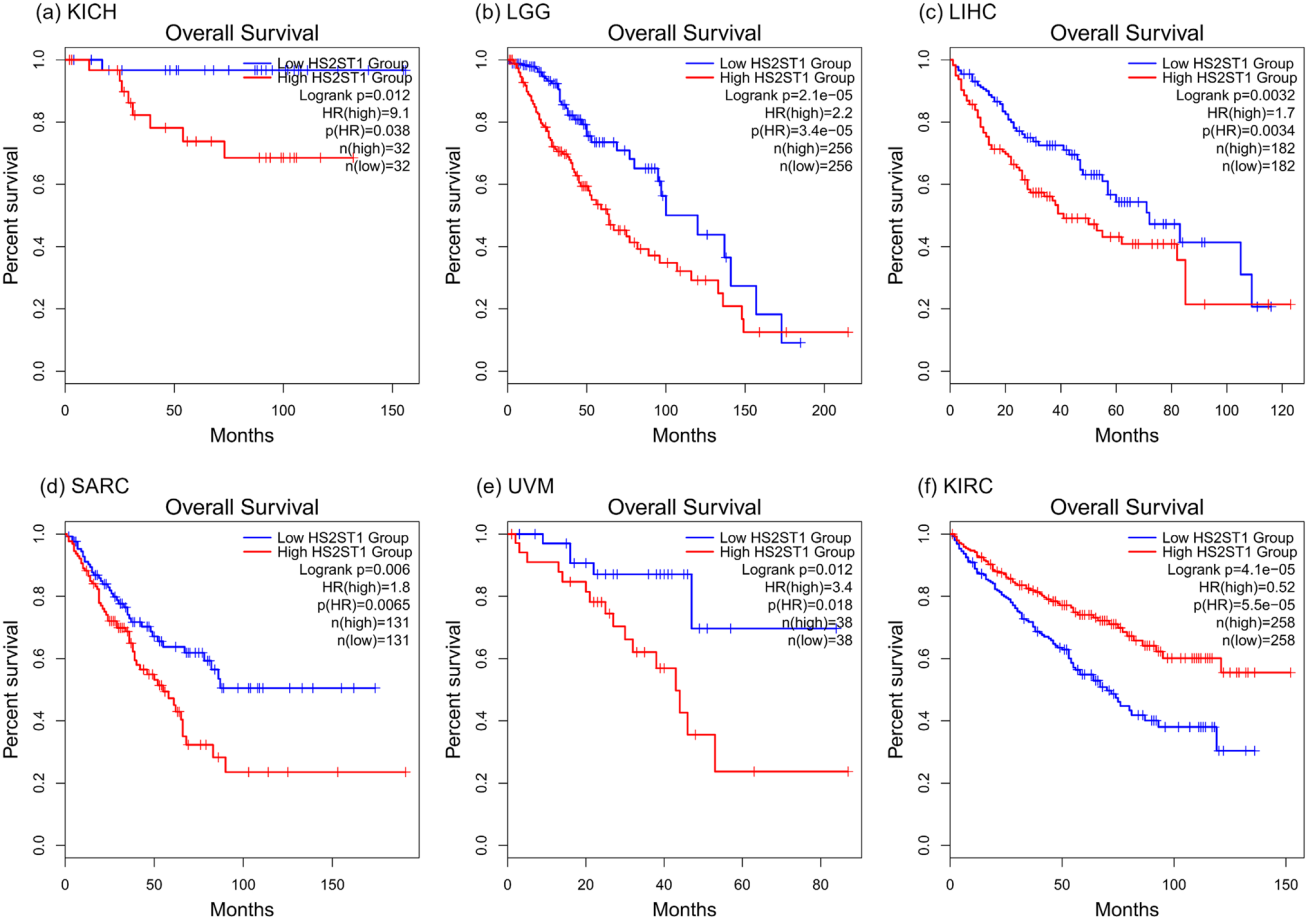
Prognostic impact of *HS2ST1* expression in TCGA cancer types. KM analysis and log-rank tests were conducted using the medians as the GEPIA2 cutoff points. Only cancers exhibiting significant differences (*P < 0.05) are shown. **a** KICH, kidney renal clear cell carcinoma, **b** LGG, brain lower grade glioma, **c** *LIHC* liver hepatocellular carcinoma; **d** *SARC* sarcoma, **e** *UVM* uveal melanoma, **f** *KIRC* kidney renal clear cell carcinoma. *HR* hazard ratio

We examined whether *HS2ST1* expression was associated with prognosis in various cancers. Survival analysis of GEPIA2 data, using median values as cutoffs, revealed that *HS2ST1* expression levels were prognostic in six cancer types (Fig. 2). Patients with high *HS2ST1* expression in kidney chromophobe (KICH), lower-grade brain glioma (LGG), LIHC, sarcoma (SARC), and uveal melanoma (UVM) had poor prognoses. High *HS2ST1* expression was associated with favorable prognosis only in patients with kidney renal clear cell carcinoma (KIRC). Thus, *HS2ST1* expression correlated with poor prognosis in patients with certain tumors. Because LIHC is a highly malignant tumor associated with high mortality, we also validated the prognostic role of *HS2ST1* expression in HCC.

### Prognostic value of HS2ST1 status in various HCC cohorts

**Fig. 3:**
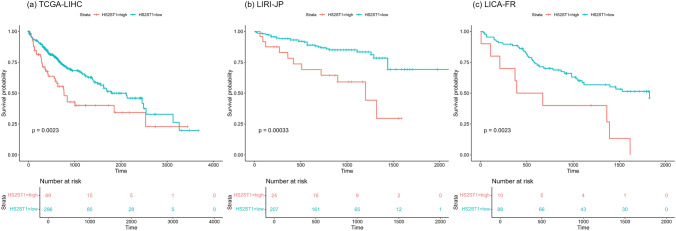
Prognostic impacts of *HS2ST1* expression in the three HCC cohorts. KM analysis and log-rank tests were conducted using the optimal cutoff points determined by maximally selected rank statistics for the **a** TCGA-LIHC, **b** LIRI-JP, and **c** LICA-FR cohorts

We explored whether *HS2ST1* mRNA expression levels predicted the prognosis of HCC patients in the TCGA-LIHC, ICGC-LIRI-JP, and ICGC-LICA-FR cohorts (Table 1). We utilized optimal cutoffs of *HS2ST1* expression levels identified by maximally selected rank statistics to maximize statistical power and flexibility afforded by the various datasets. In the TCGA-LIHC cohort, 69 primary tumor samples exhibited high *HS2ST1* expression and 296 exhibited low expression. Survival analysis revealed a significant association between *HS2ST1* expression levels and overall survival (*P* = 0.0023) (Fig. 3a). Lower *HS2ST1* expression was protective; higher expression correlated with shorter survival of HCC patients. In the LIRI-JP and LICA-FR cohorts, higher *HS2ST1* expression correlated significantly with poorer overall survival (*P* = 0.00033 and *P* = 0.0023, respectively) (Figs. 3b and c). Thus, *HS2ST1* expression levels were prognostically significant across different HCC cohorts and might aid patient stratification.

**Table 1:** Clinical and pathological information across three cohorts

Characteristics	Number of patients		
	TCGA-LIHC	ICGC-LIRI-JP	ICGC-LICA-FR
Total number of patients	365	231	98
Age (years)			
< 65	216 (59.2%)	82 (35.5%)	41 (41.8%)
≥ 65	149 (40.8%)	149 (64.5%)	57 (58.2%)
Gender			
Female	119 (32.6%)	61 (26.4%)	22 (22.4%)
Male	246 (67.4%)	170 (73.6%)	76 (77.6%)
Number of death	131 (35.9%)	42 (18.2%)	48 (49.0%)
Mean survival time (days) (95% CI)	811.9 (737.2–886.6)	812.3 (758.1–866.6)	1012.8 (888.7–1136.9)
Median survival time (days)	596	780	959
Tumor pathology	*Histologic grade*	*Edmondson tumor grade*	*Edmondson/WHO differentiation*
	G1: 55, G2: 175, G3: 118, G4: 12, NA: 5	I: 20 I–II: 10 I–III: 1 II: 123 II-I: 1 II–III: 35 III: 21 IV: 1 NA: 19	I: 2 II: 27 III: 50 IV: 16 Poor: 1 Moderate: 1 Well: 1
	*Tumor stage*		
	i: 170, ii: 84, iii: 83, iv: 4, Not reported: 24		
	*Pathologic T*		
	T1: 180, T2: 91, T3: 78, T4: 13, TX: 1, NA: 2 *Pathologic N*		
		*TNM by LCSGJ*^a^ *(at diagnosis﻿)*	*TNM 7th Edition*
	N0: 248, N1: 4, NX﻿: 112, NA: 1	1: 37 2: 104 3: 71 4: 19	T1N0M0: 24 T2N0M0: 40 T3aN0M0:17 T3bN0M0: 14 NA: 3
	*Pathologic M*		
	M0: 263, M1: 3, MX: 99		

*NA* not available

^a^*LCSGJ* Liver Cancer Study Group of Japan

### Protein–protein interaction, DEG, and gene set enrichment analyses

**Fig. 4:**
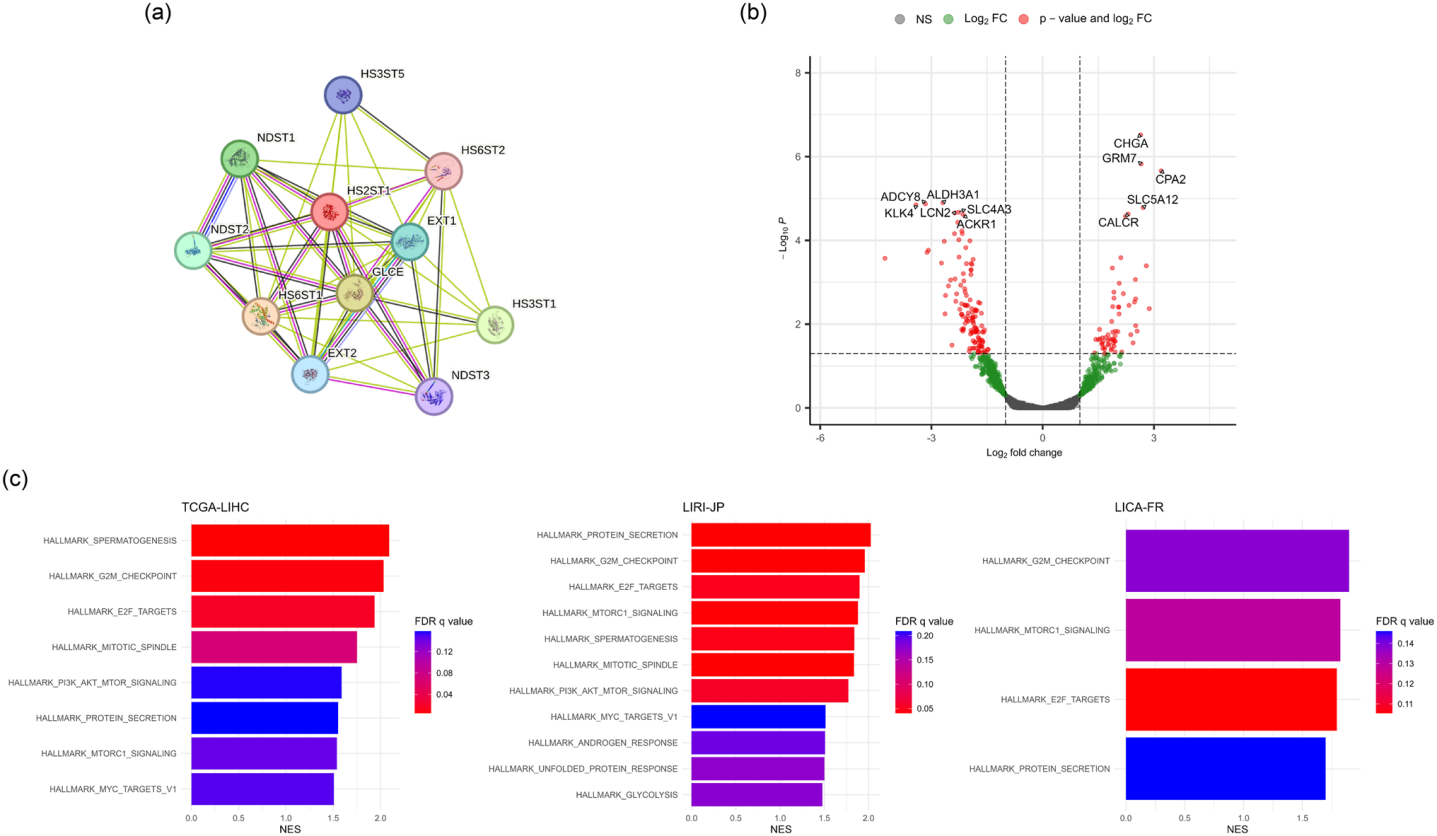
Functional analysis of the protein network, DEGs, and enriched gene sets associated with *HS2ST1* expression. **a** Protein–protein interaction analysis using the STRING database revealed a network of proteins associated with HS2ST1. **b** Volcano plot highlighting DEGs between the high- and low-*HS2ST1* expression groups with adjusted *P* values < 0.05 and |log_2_-fold changes (LFCs)| ≥  1. **c** Functional GSEA comparing the high- and low-*HS2ST1* expression groups; highlighted pathways were enriched in the high-*HS2ST1* expression group. *FDR* false discovery rate, *NES* normalized enrichment score

To explore whether HS2ST1 expression levels affected prognosis, we used STRING database to investigate protein–protein interactions involving HS2ST1 and compared DEGs of patients exhibiting high and low *HS2ST1* expression. HS2ST1 interacts with various enzymes that both biosynthesize and modify HSPGs, including exostosin glycosyltransferase 1 (EX1), glucuronic acid epimerase (GLCE), *N*-deacetylase/*N*-sulfotransferase (NDST), heparan sulfate-glucosamine 3-sulfotransferase (HS3ST), and heparan sulfate 6-*O*-sulfotransferase (HS6ST) (Fig. 4a). These proteins are known to interact with HS2ST1 but provide limited information in interpreting the prognostic role of HS2ST1 in HCC.

To further explore how high HS2ST1 expression levels might contribute to the poor prognosis of HCC patients, we identified DEGs in the TCGA-LIHC cohort and enriched gene sets in the high-*HS2ST1* expression group. DEG analysis revealed that genes encoding chromogranin A (CHGA), GRM7 glutamate metabotropic receptor 7 (GRM7), CPA2 carboxypeptidase A2 (CPA2), calcitonin receptor (CALCR), and solute carrier family 5 member 12 (SLC5A12) were more highly expressed. Conversely, genes encoding ADCY8 adenylate cyclase 8 (ADCY8), kallikrein-related peptidase 4 (KTP4), lipocalin 2 (LCN2), aldehyde dehydrogenase 3 family member A1 (ALDH3A1), atypical chemokine receptor 1 (ACKR1), and solute carrier family 4 member 3 (SLC4A3) exhibited lower expression in the high-HS2ST1 expression group than in the low-*HS2ST1* expression group (Fig. 4b).

Next, GSEA was performed using the hallmark gene set (Fig. 4c). The G_2_M checkpoint, E2F target, protein secretion, and mTORC1 signaling pathways were significantly enriched in the high-*HS2ST1* expression group compared with the low-*HS2ST1* expression group across all three cohorts. The activities of the unfolded protein response and glycolysis pathways were significantly enhanced in the LIRI-JP cohort exhibiting high *HS2ST1* expression compared with the low-*HS2ST1* expression group.

### Immune cell infiltration and *HS2ST1* expression levels

**Fig. 5:**
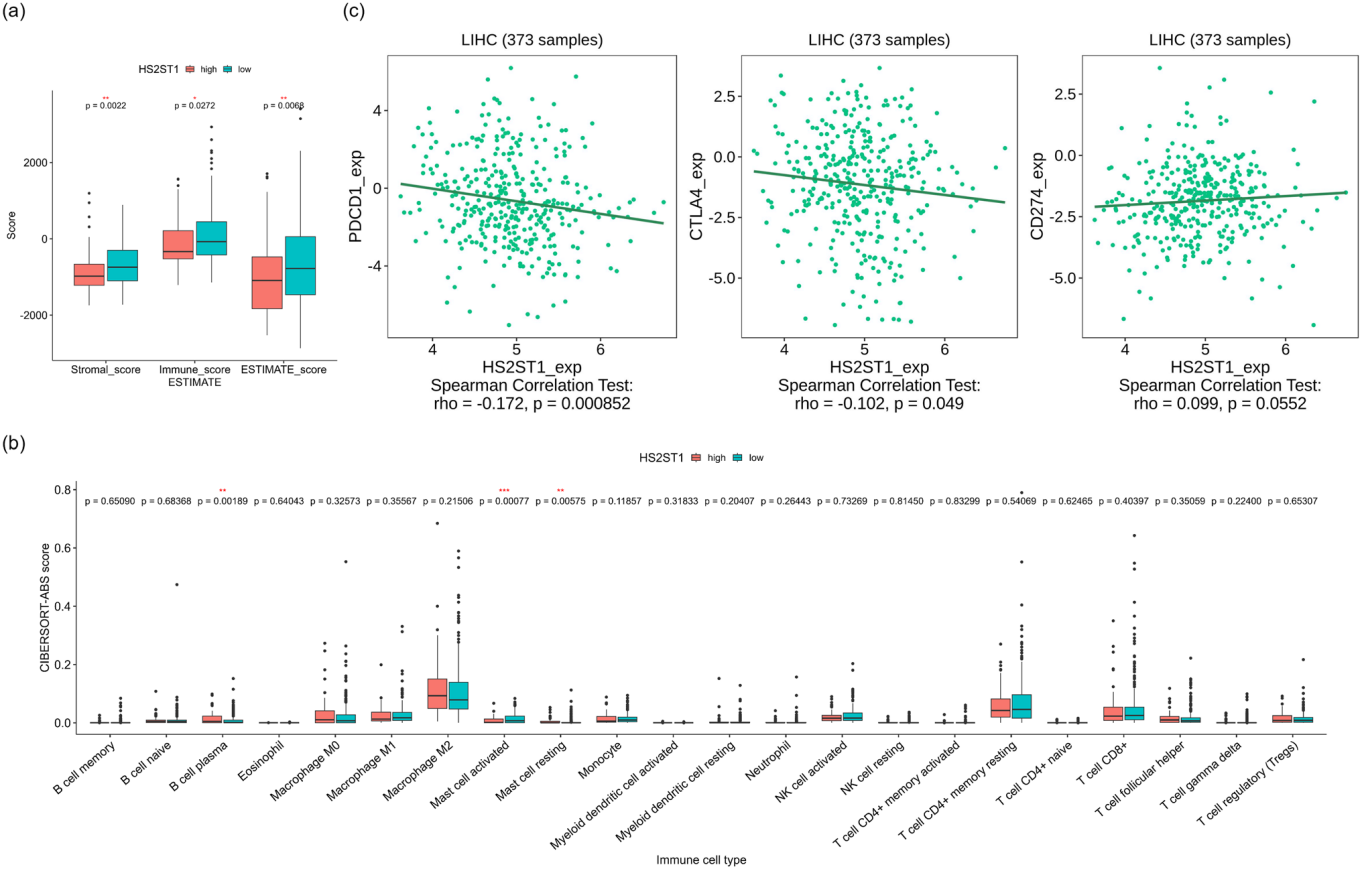
Association of the tumor immune microenvironment with *HS2ST1* expression in the TCGA-LIHC cohort. **a** Stromal, immune, and ESTIMATE scores determined by the ESTIMATE algorithm for the high- and low-*HS2ST1* expression groups (*P < 0.05). **b** Infiltration levels of various immune cell types according to *HS2ST1* expression status, as revealed by the CIBERSORT algorithm (*P < 0.05). **c** Correlations between the expression of HS2ST1 and immune checkpoint proteins, as revealed by the TISIDB (*P < 0.05)

Hs2st1 facilitates immune cell infiltration in mouse models of airway inflammation (Axelsson et al. 2012; Ge et al. 2018). Therefore, we explored whether HS2ST1 status affected the tumor microenvironment. The stromal (*P* = 0.0022) and immune scores (*P* = 0.0272) of the ESTIMATE algorithm were lower in the high-*HS2ST1* expression group. The ESTIMATE score, which combines stromal and immune scores to derive a measure of tumor purity, indicated fewer nontumor cells in the high-*HS2ST1* expression group (*P* = 0.0068) (Fig. 5a).

We used CIBERSORT software to identify specific immune cell populations associated with *HS2ST1* expression (Fig. 5b). The high-*HS2ST1* expression group contained fewer activated mast cells but more plasma B cells and resting mast cells compared with the low-*HS2ST1* expression group. Next, we used the TISIDB database to reveal correlations between *HS2ST1* expression levels and those of key immune checkpoint proteins (Fig. 5c). *HS2ST1* expression levels were weakly negatively correlated with those of PD-1 (PDCD1) (rho = − 0.172, *P* = 0.000852) and CTLA-4 (rho = − 0.102, *P* = 0.049). The correlation between *HS2ST1* expression and that of CD274 (also known as PD-L1) was marginally nonsignificant (rho = 0.099, *P* = 0.0552). These findings underscore the complex interplay between *HS2ST1* expression levels and the HCC microenvironment.

## Discussion

Sulfation of HSPG heparan chains critically mediates interactions among growth factors, cytokines, and immune cells. The roles of HS2ST1 in cancer and the overall immune landscape remain poorly understood. In this study, we used TCGA datasets to identify tumors in which *HS2ST1* was highly expressed or HS2ST1 levels were prognostic. The role of HS2ST1 status as a negative prognostic factor in TCGA-LIHC was validated in two ICGC HCC patient cohorts. High HS2ST1 expression in HCC tumors was associated with cell signaling that enhanced cell proliferation and protein secretion, as well as changes in immune cell components in the tumor microenvironment.

Glypican-3 expression levels are higher in HCC than in surrounding normal tissues; thus, glypican-3 is a HSPG commonly targeted by biologics such as chimeric antigen receptors, bispecific antibodies, and immunotoxins (Fu et al. 2023). *HS2ST1* expression was higher in tumor tissues compared with normal tissues in patients with diffuse large B-cell lymphomas, esophageal carcinomas, glioblastoma multiforme, pancreatic adenocarcinomas, rectal adenocarcinomas, skin cutaneous melanomas, stomach adenocarcinomas, and thymomas. Normal tissues adjacent to diffuse large B-cell lymphomas and thymomas expressed very low levels of *HS2ST1*, suggesting that HS2ST1 can serve as a cancer-specific marker in such patients. Higher *HS2ST1* expression was associated with poor prognoses in patients with kidney chromophobe syndrome, lower-grade gliomas, HCCs, sarcomas, and uveal melanomas. To extend the reliability of this evidence across cohorts of different nationalities, we evaluated the prognostic value of HS2ST1 expression levels in HCC cohorts from Japan and France. Higher HS2ST1 levels robustly predicted poorer survival.

Liver HSPGs interact with many growth factors (e.g., basic FGF, hepatocyte growth factors, platelet-derived growth factors, vascular endothelial growth factors), transforming growth factor-β and cytokines (CCL5 and CXCL12) (Lai et al. 2004, 2008). Such HSPG interactions, along with those involving growth factor receptors, facilitate growth factor retention, protect factors from degradation, and ultimately trigger tumor suppression. For example, *HS2ST1* overexpression decreased FGF-2/receptor interaction (Kumar et al. 2020), in turn reducing FGFR-MAPK signaling and invasion by MDA-MB-231 and MCF7 cells. Both HS2ST1 and syndecan-1 promoted FGFR1 endocytosis and inhibited FGFR1-Akt signaling in MCF7 cells (Kang et al. 2020). Interactions between heparan sulfate chains and proteases facilitate allosteric protease activation that promotes tumor progression. Syndecan-2 enhances pro-MMP-7 processing and the subsequent cleavage of the E-cadherin substrate, enhancing colon cancer cell migration (Sarrazin et al. 2011).

Currently, the binding specificities between sulfate groups in the heparan sulfate chains of HSPGs and ligands are not fully understood in the HCC context. Increased sulfation associated with decreased expression of sulfatase-1, an extracellular enzyme that remodels HSPGs, enhanced FGF- and HGF-mediated signaling, and increased HCC cell growth (Lai et al. 2004). Conversely, enhanced sulfation caused by sulfatase-2 knockdown inhibited HCC cell proliferation and migration via FGF2-ERK signaling (Lai et al. 2008). Therefore, sulfation-regulating enzymes may selectively affect ligand binding to HSPGs. Our GSEA results revealed that increased *HS2ST1* expression activated mTORC1 signaling, E2F-dependent gene expression, G_2_M checkpoint and protein secretion in three cohorts of HCC patients. Additionally, we identified DEGs associated with high *HS2ST1* expression, including several types of proteases (kallikrein-related peptidase 4 and carboxypeptidase A2), receptors (atypical chemokine receptor 1 and the calcitonin receptor), and signaling molecules (adenylate cyclase 8, chromogranin A, and lipocalin 2). HS2ST1 may regulate the expression of these proteins and thus affect HCC tumor progression. These results suggest that HS2ST1-mediated 2-*O*-sulfuration of iduronic acid residues in HCC patients promotes growth factor signaling, cell proliferation, and protein secretion. However, the DEGs identified in the TCGA-LIHC cohort were not found in the other ICGC cohorts, suggesting further studies are warranted to identify plausible partners of HS2ST1 regardless of tumor heterogeneity.

We found that tumors expressing high levels of *HS2ST1* exhibited limited stromal cell infiltration and less infiltration of activated mast cells compared with those with low *HS2ST1* expression (Fig. 5). Such suppression of the immune environment may explain the poor prognostic value of HS2ST1 status in HCC patients. Previous studies using mouse airway inflammation models also supported an immunosuppressive role for Hs2st1. Inactivation of mouse endothelial cell *Hs2st1* triggered elevated neutrophil trafficking via L-selectin-dependent rolling of neutrophils and increased binding of IL-8 and macrophage inflammatory protein-2 to endothelial cells (Axelsson et al. 2012). Leukocyte and endothelial cell *Hs2st1* deficiencies in a mouse model of allergic asthma enhanced eosinophil recruitment associated with persistent inflammation (Ge et al. 2018). Further work is needed to elucidate the interactions between immune cells and 2-*O*-sulfated uronic acid residues in heparan sulfate chains and the associations between immune checkpoint proteins and HS2ST1 in the tumor microenvironment.

Considering the therapeutic benefits provided by an understanding of how heparin pentasaccharide binds to antithrombin, further exploration of HS2ST1/ligand interactions could prove highly informative. Although our single-gene analysis provided accurate assessments of prognostic risks in independent, large patient cohorts, it remains unclear whether alterations in HS2ST1 expression directly influence disease progression or if other underlying genetic factors are involved. To address these uncertainties and efficiently elucidate the specific biological pathways involved, multi-gene analysis of the interconnected HSPG network is necessary. Our study elucidates the prognostic value of HS2ST1 status in patients with specific tumors, including HCC. *HS2ST1* expression was associated with increased signaling related to E2F-mediated gene expression, protein secretion, and mTORC1 signaling, as well as reduced immune cell infiltration. These results enhance our understanding of HS2ST1's roles in HCC and the tumor microenvironment, providing a rationale for therapeutic applications of HS2ST1.

## Data Availability

The datasets presented in this study can be found in online repositories. TCGA and GTEx datasets are available on GEPIA2. The RNASeq and clinical data for TCGA-LIHC are available on UCSC Xena Browser (http://xena.ucsc.edu/). The RNASeq and clinical data for ICGC-LIRI-JP and ICGC-LICA-FR were downloaded from the ICGC data portal (https://dcc.icgc.org/). All other data supporting the findings of this study are available within the article.
